# A systematic review of the use of an expertise-based randomised trial design

**DOI:** 10.1186/1745-6215-16-S2-O55

**Published:** 2015-11-16

**Authors:** Jonathan Cook, Andrew Elders, Charles Boachie, Ted Bassigna, Cynthia Fraser, Doug Altman, Isabelle Boutron, Craig Ramsay, Graeme Maclennan

**Affiliations:** 1 University of Oxford, Oxford, UK; 2 University of Aberdeen, Aberdeen, UK; 3 Université Paris Descartes, Paris, France; 4 University of Glasgow, Glasgow, UK; 5 Glasgow Caledonian University, Glasgow, UK

## Background

An *expertise-based* randomised trial design, where participating health professionals only provide the intervention in which they have expertise, has been proposed to overcome challenges faced when evaluating skill based interventions. Health professionals (e.g. surgeons/therapists) often have differing levels of expertise in the interventions, conduct only one routinely and/or have strong preferences. However understanding of this design is limited.

## Aim

To systematically identify and review the use of an expertise-based trial design in the medical literature.

## Methods

A comprehensive search was carried which included searching 9 databases. Studies using an expertise-based trial design were included. Two reviewers independently screened the titles and abstracts and assessed full text reports. Data on the context, study methodology and the experience of using an expertise trial design was extracted and summarised.

## Results

In total 7476 titles and abstracts were identified leading to 43 included studies (54 articles). Interventional procedures and psychotherapy (both 40%) were the most common types of interventions. Key information relating to the expertise based design was often not reported (12 (28%) provided criteria for delivering both interventions). Most studies recruited to the target size (median [IQR]:101 [94,118] %) and had high levels of allocation compliance (92 [82,99] %).

## Discussion

The use of an expertise based trial design is growing though remains uncommon. Reporting of related methodology was particularly poor. Empirical evidence provided some support for purported benefits regarding recruitment and allocation compliance. An expertise-based trial design is an option which should be considered by trialists though its merit seems context specific.

